# Multiomics Analysis Reveals Molecular Abnormalities in Granulosa Cells of Women With Polycystic Ovary Syndrome

**DOI:** 10.3389/fgene.2021.648701

**Published:** 2021-05-18

**Authors:** Rusong Zhao, Yonghui Jiang, Shigang Zhao, Han Zhao

**Affiliations:** ^1^Center for Reproductive Medicine, Cheeloo College of Medicine, Shandong University, Jinan, China; ^2^National Research Center for Assisted Reproductive Technology and Reproductive Genetics, Shandong University, Jinan, China; ^3^Key Laboratory of Reproductive Endocrinology of Ministry of Education, Shandong University, Jinan, China; ^4^Shandong Provincial Clinical Medicine Research Center for Reproductive Health, Shandong University, Jinan, China

**Keywords:** polycystic ovary syndrome, methylome, transcriptome, metabolism, steroid biosynthesis

## Abstract

Polycystic ovary syndrome (PCOS) is the most common complex endocrine and metabolic disease in women of reproductive age. It is characterized by anovulatory infertility, hormone disorders, and polycystic ovarian morphology. Regarding the importance of granulosa cells (GCs) in the pathogenesis of PCOS, few studies have investigated the etiology at a single “omics” level, such as with an mRNA expression array or methylation profiling assay, but this can provide only limited insights into the biological mechanisms. Here, genome-wide DNA methylation together with lncRNA-miRNA-mRNA profiles were simultaneously detected in GCs of PCOS cases and controls. A total of 3579 lncRNAs, 49 miRNAs, 669 mRNAs, and 890 differentially methylated regions (DMR)-associated genes were differentially expressed between PCOS cases and controls. Pathway analysis indicated that these differentially expressed genes were commonly associated with steroid biosynthesis and metabolism-related signaling, such as glycolysis/gluconeogenesis. In addition, we constructed ceRNA networks and identified some known ceRNA axes, such as lncRNAs-miR-628-5p-CYP11A1/HSD17B7. We also identified many new ceRNA axes, such as lncRNAs-miR-483-5p-GOT2. Interestingly, most ceRNA axes were also closely related to steroid biosynthesis and metabolic pathways. These findings suggest that it is important to systematically consider the role of reproductive and metabolic genes in the pathogenesis of PCOS.

## Introduction

Polycystic ovary syndrome (PCOS) is a life-long reproductive, neuroendocrine, and metabolic disorder that affects up to 6–15% of women of reproductive age (Risal et al., 2019). Its main clinical manifestations are ovulatory dysfunction, hyperandrogenemia, and polycystic ovaries, which can lead to infertility (Fauser et al., 2012). In addition to the above reproductive disorders, PCOS is often accompanied by metabolic abnormalities, such as insulin resistance (IR). IR can increase pituitary luteinizing hormone (LH) secretion, testosterone secretion in theca cells, and P450scc activity in granulosa cells (GCs), which interferes with follicle maturation and leads to the development of PCOS (Li et al., 2019). Studies have shown that the abnormal ovarian hormone production is mainly attributed to the hypertrophy of follicular theca cells and the altered expression of key steroid biosynthesis enzymes in GCs (Aste et al., 1998).

As the most abundant cells in the ovary, GCs are closely associated with the development of oocytes and play an essential role in both normal folliculogenesis and steroidogenesis (Hummitzsch et al., 2015). Previous studies have shown that cumulus and mural GCs contribute to the process of oocyte maturation by tight regulation and controlled changes in steroid hormones in the pathogenesis of PCOS (Holesh et al., 2020). Oocytes lack the capacity to carry out some metabolic processes, such as glycolysis and amino acid uptake. They rely on GCs to deliver nutrients and remove waste. In addition, the metabolic profile of GCs is associated with the fate of their accompanying oocyte (Gioacchini et al., 2018; Yilmaz et al., 2018; Fontana et al., 2020).

Previous studies have separately screened differentially expressed mRNAs, miRNAs (DEMs) and lncRNAs (DELs) in GCs to explore the regulatory mechanism of PCOS. Few studies have indicated that genome-wide DNA methylation changes may affect the expression of different genes in PCOS ovaries, as revealed by methylated DNA immunoprecipitation (MeDIP) experiments (Yu et al., 2015; Xu et al., 2016). However, to date, no studies have been performed to identify the whole transcriptome and methylome in same GCs of women with PCOS. In this study, the lncRNA-miRNA-mRNA expression profiles and DNA methylation of GCs in PCOS were comprehensively analyzed. Our goal was to integrate multiomics data to identify differentially expressed genes (DEGs) and differentially methylated regions (DMRs) in PCOS and to construct molecular networks that could help us to better understand the etiology of PCOS.

## Materials and Methods

### Sample Selection

Five women with PCOS and five age/body mass index (BMI)-matched control subjects from the Center for Reproductive Medicine, Shandong University, Jinan, China, were included in this study. All patients gave informed consent, and the study was approved by the institutional review board of the Reproductive Hospital Affiliated to Shandong University. The definition of PCOS was based on the 2003 Rotterdam criteria (Rotterdam ESHRE/ASRM-Sponsored PCOS Consensus Workshop Group, 2004). Women considered as suffering from PCOS had at least two of the following three characteristics: polycystic ovaries on ultrasound, irregularity/absence of menses, and hyperandrogenism. Cases with congenital adrenal hyperplasia, androgen-secreting tumors, Cushing’s syndrome, thyroid disease, and hyperprolactinemia were excluded. The control group was selected from healthy women who attended the center for IVF with their husbands due to a male factor. All relevant clinical information was obtained from the Electronic Medical Records System. BMI was calculated as weight (kg)/height^2^ (m). Peripheral blood was collected on the 3rd to 5th days of the menstrual cycle to measure serum hormone levels. The levels of follicle-stimulating hormone (FSH), LH, estrogen (E2), prolactin (PRL), and testosterone (T) were measured with a chemiluminescence analyzer (Beckman Coulter, United States). Type B ultrasound was used to determine the antral follicle counts (AFC).

### Retrieval of GCs

GCs were collected from follicular fluid obtained via ultrasound-guided transvaginal oocyte retrieval after informed consent had been given by the patients who received the long gonadotropin-releasing hormone agonist protocol. Oocyte retrieval was performed 36 h after human chorionic gonadotropin (hCG) injection by transvaginal ultrasound-guided needle puncture for follicles >15 mm in diameter. At the time of oocyte retrieval, follicular fluid aspirates were collected in sterile tubes and centrifuged. GCs were isolated and purified from the follicular fluid with Ficoll-Percoll (Solarbio, Beijing, China) as previously described (Iwase et al., 2009), and then immediately stored at –80°C for further analysis.

### RNA−Seq Analysis and Quality Assessment

Total RNA was extracted using TRIzol Reagent (Invitrogen, CA, United States) and purified using an RNeasy Mini Kit (Qiagen, CA, United States). The quality of RNA was assessed using an Agilent 2100 Bioanalyzer (Agilent, Palo Alto, CA, United States). The rRNA-depleted RNA samples were further processed in accordance with the Illumina protocol (New England Biolabs, Massachusetts, United States). After cDNA synthesis, the samples were sequenced with an Illumina HiSeq 2500 using the paired-end (PE) sequencing strategy. The raw data were recorded. The overall quality of the RNA−seq data was evaluated by FastQC. Clean reads were aligned to the reference genome (Ensembl release 95, *Homo sapiens*) using TopHat2 (v2.0.14) (Kim et al., 2013) with the default parameters.

### MeDIP-Seq Library Construction

MeDIP is a method for immunoprecipitating the methylated portion of the genome using an antibody capable of recognizing 5mC (Wilson and Beck, 2016). Following the manufacturer’s instructions, MeDIP was performed to analyze genome-wide methylation using the Zymo Research DNA Methylation IP Kit (Cat #D5101; Zymo Research, CA, United States). Immunoprecipitated DNA was PCR-amplified, purified, quantified, and sequenced on the Illumina HiSeq 2500 platform. MeDIP−seq reads were mapped to the human genome using BWA software (Li et al., 2012). MACS2 was used to call peaks. To study the DNA methylation differences between two groups, DMRs were identified using the Cummerbund (Trapnell et al., 2012) and ChIPpeakAnno (Zhu et al., 2010) packages in R. Briefly, DMRs were assigned to genomic regions based on gene annotations available from JGI and in-house repeat annotation in GFF3 format. The following gene regions were included: 3’UTR, 5’UTR, promoter, coding DNA sequence (CDS), intron, upstream 1 kb, and downstream 1 kb.

### Screening and Clustering Analysis of Differentially Expressed mRNAs, DELs, and DEMs

Data preprocessing and follow-up analysis were performed in the R programming environment (version 3.6.1), and Bioconductor packages were applied for the analysis of DEGs. The lists of DEGs, DELs, and DEMs between controls and PCOS cases were generated using the edgeR package (version 3.32.0) (Robinson et al., 2010). To normalize the raw data, log-fold change | (logFC)| > 1.2 (mRNA and miRNA), | (logFC)| > 2.0 (lncRNA) and *p*-value <0.05 were considered to indicate statistically significant differences between the PCOS and control groups. To generate an overview of the lncRNA, miRNA and mRNA expression profiles and compare them between the two groups, hierarchical clustering analysis was performed based on the expression levels of all transcripts and significantly differentially expressed transcripts using the pheatmap R package based on Euclidean distance.

### lncRNA, miRNA, and mRNA Prediction and Coexpression Network Construction

The miRNA target genes were predicted using the prediction results of the TargetScan and miRcode (Jeggari et al., 2012) databases. The potential target genes transcribed within a 10-kb region upstream or downstream of the lncRNAs were paired and predicted using the UCSC Genome Browser^1^ (Song et al., 2019). The expression of differentially expressed mRNAs, DEMs and DELs was analyzed by Pearson’s correlation coefficient using the stats and pcaPP R packages. The miRNA-mRNA network and the lncRNA-mRNA coexpression network were constructed based on analysis of the correlations among the differentially expressed mRNAs, DEMs, and DELs. A p-value of <0.05 for the miRNA-mRNA network and one of <0.01 for the lncRNA-mRNA network were considered statistically significant. The target genes that overlapped with the DEGs were then identified and used to construct the miRNA-mRNA network and lncRNA-mRNA network using Cytoscape software (version 3.8.1) (Saito et al., 2012).

### Functional Enrichment Analysis

The Kyoto Encyclopedia of Genes and Genomes (KEGG) pathway enrichment analysis in each module and network was conducted using the Database for Annotation, Visualization and Integrated Discover (DAVID). DEGs and enriched pathways were mapped using KEGG pathway annotation with KOBAS3.0^2^. The top 10 KEGG pathways were selected and ranked by the enrichment factor. To perform literature-based functional analysis, a total of 370 follicle development- and 437 steroid metabolism-related genes were obtained from the Ovarian Kaleidoscope Database (OKDB)^3^. To identify key transcription factors (TFs), a total of 1496 human TFs were obtained from the Human Protein Atlas Database (HPA)^4^. Subsequently, the Venn diagram tool was used to help identify the common genes that were the focus of this work.

### Statistical Analysis

Regarding the clinical characteristics of PCOS patients and controls, quantitative variables are expressed as the mean ± SD. *P* < 0.05 was considered significant. The clinical data analyses were performed with Statistical Package for Social Science (SPSS 25.0; IBM Corp, Armonk, NY, United States).

## Results

### Clinical Features

Table 1 presents the basic statistics of both PCOS and control subjects regarding the most important characteristics, such as FSH, LH, E_2_, T, P, PRL, and AFC levels, as well as age and BMI. Significant differences between the two groups were found for LH, T, and AFC, all of which had higher levels in PCOS cases (*P* < 0.05).

**TABLE 1:** Clinical characteristics of women with polycystic ovary syndrome and controls.

	PCOS	Control	*P*-value
Age, years	29 ± 1.0	28.4 ± 2.07	0.576
BMI, kg/m^2^	22.23 ± 1.87	22.26 ± 2.00	0.984
FSH, IU/L	6.53 ± 0.87	6.46 ± 0.51	0.874
LH, IU/L	12.20 ± 3.59	4.32 ± 2.43	0.004
E_2_, pg/ml	55.14 ± 18.34	34.35 ± 14.27	0.08
PRL, ng/ml	28.50 ± 26.10	18.31 ± 10.51	0.442
T, ng/dl	52.42 ± 8.14	15.07 ± 9.40	0.001
AFC, *n*	38.0 ± 16.40	17.00 ± 1.41	0.045

*Data are presented as the mean ± SD. BMI, body mass index; LH, luteinizing hormone; FSH, follicle-stimulating hormone; T, testosterone; AFC, antral follicle counts.*

### Differential Expression Analysis

**FIGURE 1:**
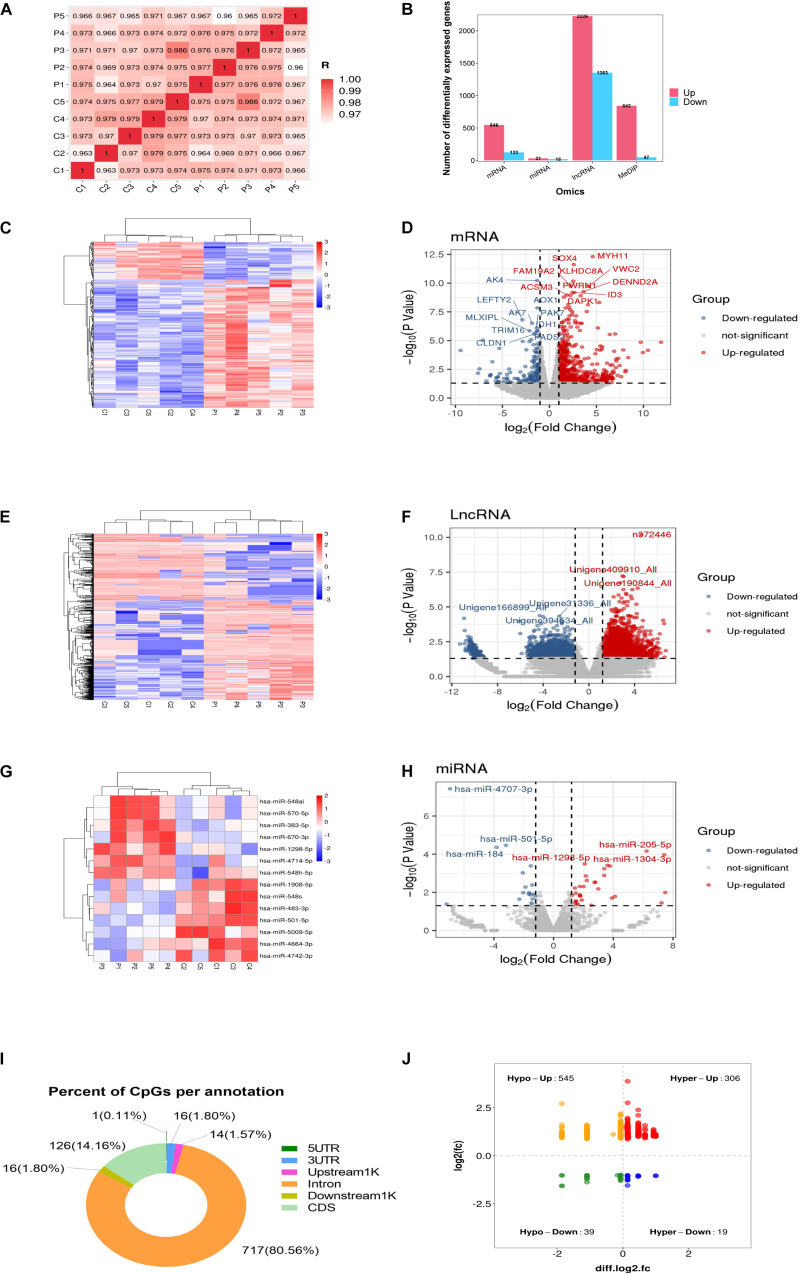
Global differentially expressed mRNAs, lncRNAs, miRNAs, and differentially methylated regions (DMRs) identified in PCOS and control granulosa cells. **(A)** Correlation heatmap between PCOS and control samples. **(B)** The numbers of differentially expressed mRNAs, differentially expressed lncRNAs (DELs) and differentially expressed miRNAs (DEMs). **(C)** Hierarchical clustering presentation of DEGs in the PCOS and control groups. **(D)** Volcano plot of DEGs in the PCOS and control groups. **(E)** Hierarchical clustering presentation of DELs in the PCOS and control groups. **(F)** Volcano plot of DELs in the PCOS and control groups. **(G)** Hierarchical clustering presentation of DEMs in the PCOS and control groups. **(H)** Volcano plot of DEMs in the PCOS and control groups. **(I)** The distribution of DMRs. **(J)** The correlation of DMR-associated genes and mRNA expression.

To identify the DEGs, GCs from five healthy women and five women with PCOS were studied. As indicated in Figure 1A, a correlation plot was used to determine the correlation between samples and to verify the homogeneity between biological replicates. As presented in the histogram in Figure 1B, 669 mRNAs, 49 miRNAs and 3579 lncRNAs were differentially expressed between the PCOS and control groups. Among them, 546 and 123 mRNAs, 31 and 18 miRNAs, and 2226 and 1353 lncRNAs were upregulated and downregulated in PCOS, respectively. Hierarchical clustering heatmaps of the differentially expressed RNAs are shown in Figures 1C,E,G. All differentially expressed mRNAs, DEMs and DELs are listed in Supplementary Table 1. The volcano plots showed the differential expression of mRNAs, miRNAs and lncRNAs between the PCOS group and control group (Figures 1D,F,H). DNA methylation analysis of the MeDIP-seq data showed 890 CpG sites that were differentially methylated in PCOS GCs compared with control GCs (Supplementary Table 1). In terms of the gene structures associated with the CpG sites, the proportions of CDS, intron, downstream 1 kb, upstream 1 kb, 3’UTR, and 5’UTR were 126 (14.16%), 717 (80.56%), 16 (1.8%), 14 (1.57%), 16 (1.8%), and 1 (0.11%), respectively (Figure 1I). We identified 545 hypomethylated and upregulated genes, 19 hypermethylated and downregulated genes, 306 hypermethylated and upregulated genes, and 39 hypomethylated and downregulated genes by integrating the DNA methylation and gene expression data (Figure 1J). Moreover, the chromosomal locations of the DMRs were examined, and they were found to be present on all chromosomes except the Y chromosome (Supplementary Figure 1).

### Functional Enrichment Analysis of DEGs, DELs, DEMs, and DMR-Associated Genes

**FIGURE 2:**
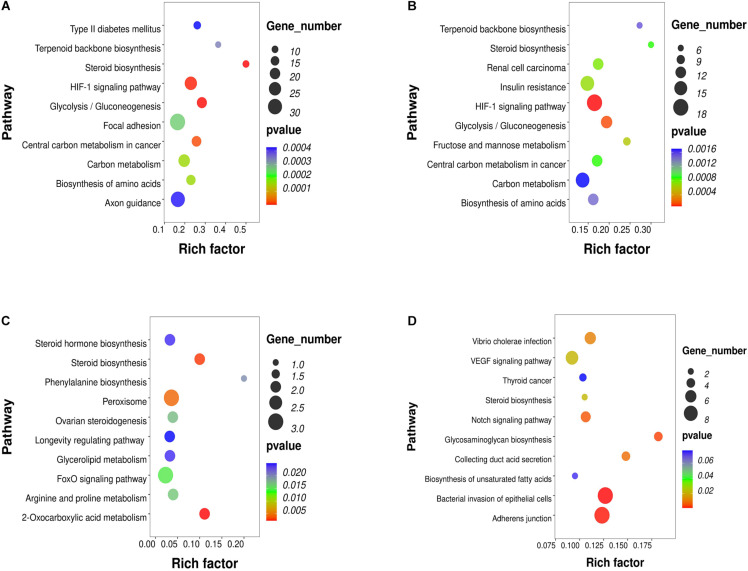
KEGG pathway analysis of RNA-seq and methylation results in PCOS and control GCs. The top 10 affected biofunctions are grouped by disease. **(A)** Enriched KEGG pathways of DEGs. **(B)** Enriched KEGG pathways of DELs. **(C)** Enriched KEGG pathways of DEMs. **(D)** Enriched KEGG pathways of DMRs.

To investigate the key pathways, the DEGs, DELs, DEMs and DMR-associated genes were evaluated and compared in terms of potential functional pathways in the KEGG database (Supplementary Table 2). As shown in Figure 2A, the results revealed that the DEGs were mainly involved in steroid biosynthesis and many metabolism-related pathways, such as type II diabetes mellitus, glycolysis/gluconeogenesis, carbon metabolism, biosynthesis of amino acids, HIF-1 signaling. Combining the known genes with human TFs, we found that the expression of FOXA1, HIF3A, and STMN1 was upregulated in PCOS GCs (Supplementary Figure 2A). The most significantly enriched biological functions of these TFs were the TGF-beta signaling pathway, IL-17 signaling pathway, endocrine resistance, human T-cell leukemia virus 1 infection, estrogen signaling pathway, Toll-like receptor signaling pathway, IR, GnRH secretion and TNF signaling pathway (Supplementary Figure 2B). For the known genes related to follicle development and steroid metabolism, AMH, FSHR, ESR2, DDX4, and SMAD9 expression was upregulated in the GCs of PCOS patients, while INHA, SOD2, and CYP11A1 expression was downregulated (Supplementary Figures 2C,D). All these functions and pathways have been proven to be closely correlated with the pathogenesis of PCOS.

To further study the role and potential mechanisms of DELs, we identified 124 of their target mRNAs. KEGG analysis identified a total of 40 significantly enriched pathways, including metabolic pathways and steroid biosynthesis (Figure 2B). Notably, the metabolic pathways included carbon metabolism, biosynthesis of amino acids, IR, HIF-1 signaling pathway, terpenoid backbone biosynthesis and glycolysis/gluconeogenesis. Analysis of DEM target genes also identified a number of pathways. Further investigation by KEGG revealed that these miRNAs participated in the regulation of metabolism and steroid synthesis, such as 2-oxocarboxylic acid metabolism, glycerolipid metabolism, arginine and proline metabolism, the FoxO signaling pathway, ovarian steroidogenesis and steroid hormone biosynthesis (Figure 2C). The DMR-associated genes were also involved in metabolic pathways and steroid pathways. Specifically, these genes were associated with glycosaminoglycan biosynthesis, collecting duct acid secretion, bacterial invasion of epithelial cells, adherens junction, steroid biosynthesis, biosynthesis of unsaturated fatty acids and the notch signaling pathway (Figure 2D). Notably, all four omics enrichment analyses identified the steroid biosynthesis pathway. In addition, all three RNA omics analyses revealed the enrichment of metabolic pathways, which are key players in steroidogenesis by acting as a source of energy and substrate for steroid production. These multiomics enrichment results suggest a common etiology of abnormal metabolism and abnormal ovarian steroid formation in GCs of women with PCOS.

### Construction of a lncRNA-miRNA-mRNA ceRNA Network

**FIGURE 3:**
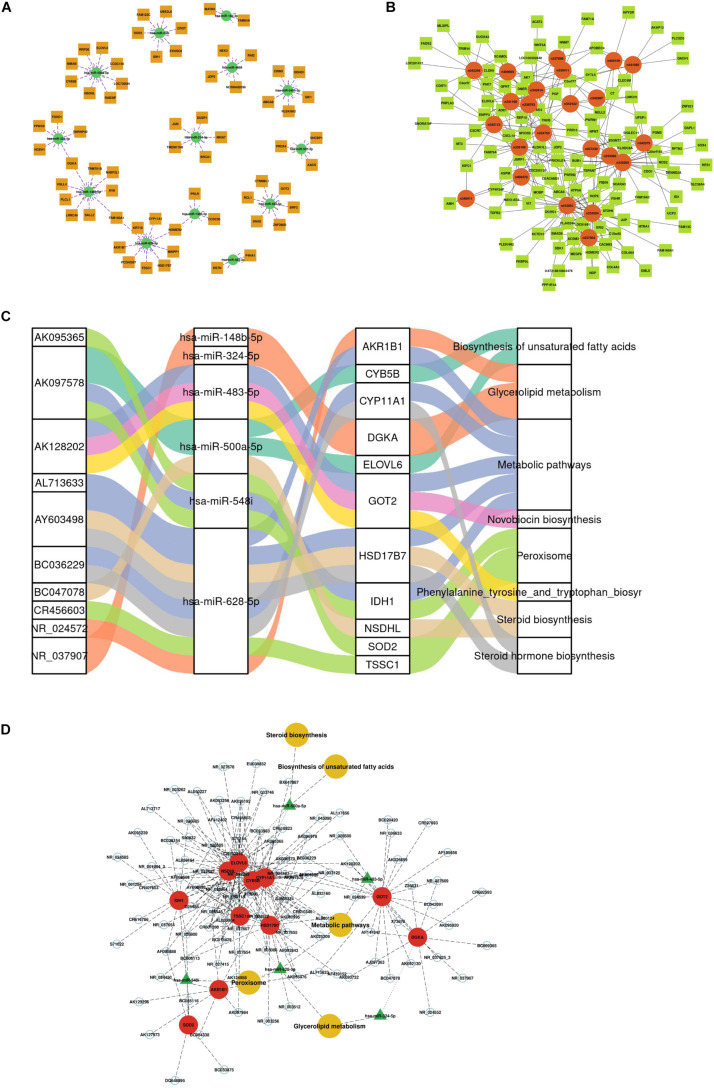
Construction of the competing endogenous (ceRNA) regulatory network. **(A)** miRNA-mRNA interaction network. **(B)** lncRNA-mRNA coexpression network. **(C)** Sankey diagram of integrative network analysis of multi-RNA-seq data. **(D)** ceRNA interaction network of miRNA-mRNA-lncRNA interactions. This plot shows the potential regulatory linkage of different RNAs and biological pathways. The four modules represent lncRNAs, miRNAs, mRNAs, and pathways.

According to the predicted correlations among lncRNAs, miRNAs and mRNAs, a competing endogenous RNA (ceRNA) network was constructed using ceRNA mechanism analysis. The miRNA-mRNA coexpression network was constructed based on the correlation analysis between the DEGs and DEMs. A total of 67 differentially expressed target genes were predicted for 13 DEMs, which were used to construct the miRNA-mRNA coexpression network (Figure 3A). This network included 10 interactions and was associated with metabolic pathways, as determined by searching the KEGG database. The interactions included hsa-miR-548i-SOD2/IDH1, hsa-miR-500a-5p-NSDHL, hsa-miR-483-5p-GOT2, and hsa-miR-214-5p-BRCA1/MKI67. Among all DEM interactions in this regulatory network, FOXO1-hsa-miR-324-5p to DGKA-hsa-miR-148b-5p, FAM160A1-hsa-miR-628-5p, and HOMER2-hsa-miR-130b-5p-PRLR were revealed to represent continuous network connections. Similar to the miRNA-mRNA network, the lncRNA-mRNA coexpression network was constructed based on analysis of the correlation between the DELs and DEGs. In total, 34 lncRNAs and 112 mRNAs involved in 326 interactions were selected to generate the network map (Figure 3B).

There were 217 nodes in the ceRNA network, which consisted of 79 lncRNAs, 6 miRNAs and 11 mRNAs, forming 8 pathways (Supplementary Table 3 and Figure 3C). The top five pathways of lncRNAs, miRNAs and mRNAs are displayed in Figure 3D, indicating the important biological significance of these molecules. KEGG pathway analysis was also performed to determine the involvement of coexpressed genes in different biological pathways. Five pathways overlapped with the enriched genes in the integrated ceRNA network, namely, glycerolipid metabolism, metabolic pathways, biosynthesis of unsaturated fatty acids, steroid biosynthesis, and peroxisome. For example, the AY603498-hsa-miR-628-5p-CYP11A1 and BC036229-hsa-miR-628-5p-HSD17B7 ceRNA axes, which contribute to steroid hormone biosynthesis, were downregulated in PCOS. The AK097578-hsa-miR-548i-IDH1 and AK128202-hsa-miR-483-5p-GOT2 networks were also identified to be associated with metabolic pathways. These analyses identified coexpressed genes that were associated with PCOS development.

## Discussion

In the present study, we systematically investigated the differences in the mRNA-miRNA-lncRNA transcriptome and methylation modifications in control and PCOS GCs. Previous studies have focused on only single methylation modifications (Xu et al., 2016; Sagvekar et al., 2019) or single transcriptomics (Jones et al., 2015; Lan et al., 2015) in GCs. In addition, there were some multiomics studies on whole blood (Li et al., 2017), follicular fluid (Naji et al., 2018) and adipose tissue (Kokosar et al., 2016; Pan et al., 2018). However, few studies have performed multiomics analyses of GCs in patients with PCOS. Although many factors have been proven to play important roles in PCOS development in recent decades, no multiomics study has been performed in ovarian GCs. In this study, we systematically investigated control and PCOS ovarian GC mRNA-miRNA-lncRNA-DNA profiles and their potential regulatory networks.

In fact, some studies have shown that in GCs of PCOS patients, there is notable disruption of the entrainment of hormones and metabolic rhythms during the menstrual cycle (Wawrzkiewicz-Jalowiecka et al., 2020). The interaction of glucose/lipid metabolism and steroid synthesis shows obvious effects on both the development and the clinical manifestations of PCOS, mainly by increasing androgen availability, changing the function of GCs and disrupting follicle development (Pasquali et al., 2006). Follicle development depends on the synchronization of oocyte maturation and GC proliferation and differentiation. At the same time, the maturation of oocytes relies on the steroids and nutrients provided by GCs (Sutton-McDowall et al., 2010). From the perspective of follicle development, some of the DEGs identified in this study were related to androgen excess, impaired ovulation, and oxidative stress. Examples of such DEGs include CYP11A1, HSD17B7, and FOXO1, which have been identified to participate in the occurrence and development of PCOS (Sagvekar et al., 2019). PCOS is also characterized by IR and hyperinsulinemia. In PCOS, we also identified the involvement of some metabolic genes, such as IRS1 and INSR, showing increased expression, and IDH1 and GOT2, showing decreased expression at the transcriptional level, which may confer a genetic predisposition to developing this condition (Feng et al., 2015). Of note, TF analysis showed a higher level of the ZBTB16 gene in PCOS GCs, which is consistent with the findings of recent PCOS susceptibility gene studies. A large-scale genome-wide meta-analysis of PCOS patients of European ancestry suggests that a variant at the ZBTB16 locus was strongly associated with ovulatory dysfunction and polycystic ovarian morphology (Day et al., 2018). The SNP rs1784692 in the ZBTB16 gene was associated with PCOS and BMI levels in Han Chinese women (Yang et al., 2020).

PCOS is a complex and heterogeneous condition that results from the interaction of diverse genetic and environmental factors (Rosenfield and Ehrmann, 2016). In our study, we identified some key common enriched pathways, including glycolysis/gluconeogenesis, steroid biosynthesis, and IR, in the KEGG analysis of lncRNA-miRNA-mRNA interactions and DMR-associated genes. By constructing a ceRNA network, we also observed that the most highly enriched ceRNA axes might play a role in regulating metabolic pathways and steroid biosynthesis in the development of PCOS. Among them, both lncRNA-miR-628-5p-CYP11A1 and lncRNA-miR-628-5p-HSD17B7 ceRNA regulatory axes are associated with steroid hormone biosynthesis and metabolic pathways. A differential expression study showed that an increase in miR-628-5p serum levels at 20 weeks of gestation was observed in women who developed severe preeclampsia (Martinez-Fierro et al., 2019). In this study, the miR-628-5p-CYP11A1/HSD17B7 network was downregulated in PCOS GCs. A randomized clinical trial showed that frozen embryo transfer resulted in an increased risk of preeclampsia in twin pregnancy in women with PCOS (Zhang et al., 2018). Other preliminary evidence described that women with PCOS and the highest maternal testosterone levels in the early second trimester had the highest risk of developing preeclampsia (Valdimarsdottir et al., 2020). A transcriptome study showed that HSD17B7 and CYP11A1 expression was repressed in DHT-treated ovaries and that the dysregulation of HSD17B7 and CYP11A1 expression was associated with the biosynthesis and metabolism of steroids and cholesterol and lipids (Salilew-Wondim et al., 2015). Therefore, miR-628-5p may be an important hub regulator of HSD17B7 and CYP11A1 and may increase the risk of pregnancy complications by affecting steroid hormone biosynthesis and metabolic pathways in PCOS patients.

We also observed a downregulated axis consisting of lncRNAs-miR-483-5p and GOT2 associated with metabolic pathways in the ceRNA network. A previous study reported that miR-483-5p expression was significantly decreased in cumulus cells of PCOS patients (Shi et al., 2015). Other miRNA expression profiles revealed that miR-483-5p can regulate Notch3/MAPK3 expression (Xu et al., 2015) and progesterone concentrations (Sang et al., 2013) in cumulus GCs and follicular fluid of PCOS patients. Although few studies have focused on the function of GOT2 in PCOS, an important conclusion was reached by Yang et al. (2015), who found that GOT2 participates in mitochondrial metabolism through acetylation (Borst, 2020). Moreover, miR-483-5p is associated with future onset of both diabetes and cardiovascular disease (Gallo et al., 2018) and increases hepatic LDL receptor levels by inhibiting PCSK9 production (Dong et al., 2020). According to these results, it can be speculated that miR-483-5p may regulate GOT2 to contribute to the IR of PCOS and is worthy of further investigation.

In summary, the results show that women with PCOS have multiple transcriptional and epigenetic changes in GCs that are related to steroid hormone synthesis and metabolic pathways. Several genes and pathways, such as lncRNAs-miR-628-5p-CYP11A1/HSD17B7 and lncRNAs-miR-483-5p-GOT2, play important roles in the etiology of PCOS and may be novel candidate biomarkers or treatment targets for PCOS.

## Data Availability Statement

The datasets presented in this study can be found in online repositories. The names of the repository/repositories and accession number(s) can be found below: NCBI GEO GSE168404.

## Ethics Statement

The studies involving human participants were reviewed and approved by Institutional review board of the Reproductive Hospital Affiliated to Shandong University. The patients/participants provided their written informed consent to participate in this study.

## Author Contributions

HZ contributed to conception and design of the study. SGZ revised the manuscript. RSZ performed bioinformatics analysis and wrote the manuscript. YHJ supervised the bioinformatics analysis and edited the manuscript. All authors contributed to manuscript revision, read, and approved the submitted version.

## Conflict of Interest

The authors declare that the research was conducted in the absence of any commercial or financial relationships that could be construed as a potential conflict of interest.

## Footnotes

1 http://genome.ucsc.edu/

2 http://kobas.cbi.pku.edu.cn/kobas3/

3 http://okdb.appliedbioinfo.net/

4 https://www.proteinatlas.org/

## Supplementary Material

Supplementary Figure 1 Chromosome graph of differentially methylated regions (DMRs) between PCOS and control GCs. Different colors represent different types of DMR loci.

Supplementary Figure 2 Specific pathway analysis of differentially expressed genes (DEGs). **(A)** Heatmap of DEGs associated with transcription factor regulation. **(B)** KEGG pathways of DEGs associated with transcription factor regulation. **(C)** Heatmap of DEGs associated with follicle development. **(D)** Heatmap of DEGs associated with steroid metabolism.

Supplementary Table 1 Differentially expressed miRNA-mRNA-lncRNA-CpG-associated genes.

Supplementary Table 2 KEGG pathways of miRNA-mRNA-lncRNA-CpG-associated genes.

Supplementary Table 3 Integrated analysis of identified signature genes in the miRNA-mRNA-lncRNA network.
