# Supplementary material for: Multiomics Analysis Reveals Molecular Abnormalities in Granulosa Cells of Women With Polycystic Ovary Syndrome
Source: Front Genet. 2021 May 18;12:648701. doi: 10.3389/fgene.2021.648701 (PMC8168535; doi:10.3389/fgene.2021.648701)
Supplement: Supplementary Figure 1 — Chromosome graph of differentially methylated regions (DMRs) between PCOS and control GCs. Different colors represent different types of DMR loci. [file Data_Sheet_1.docx]

Supplementary Material


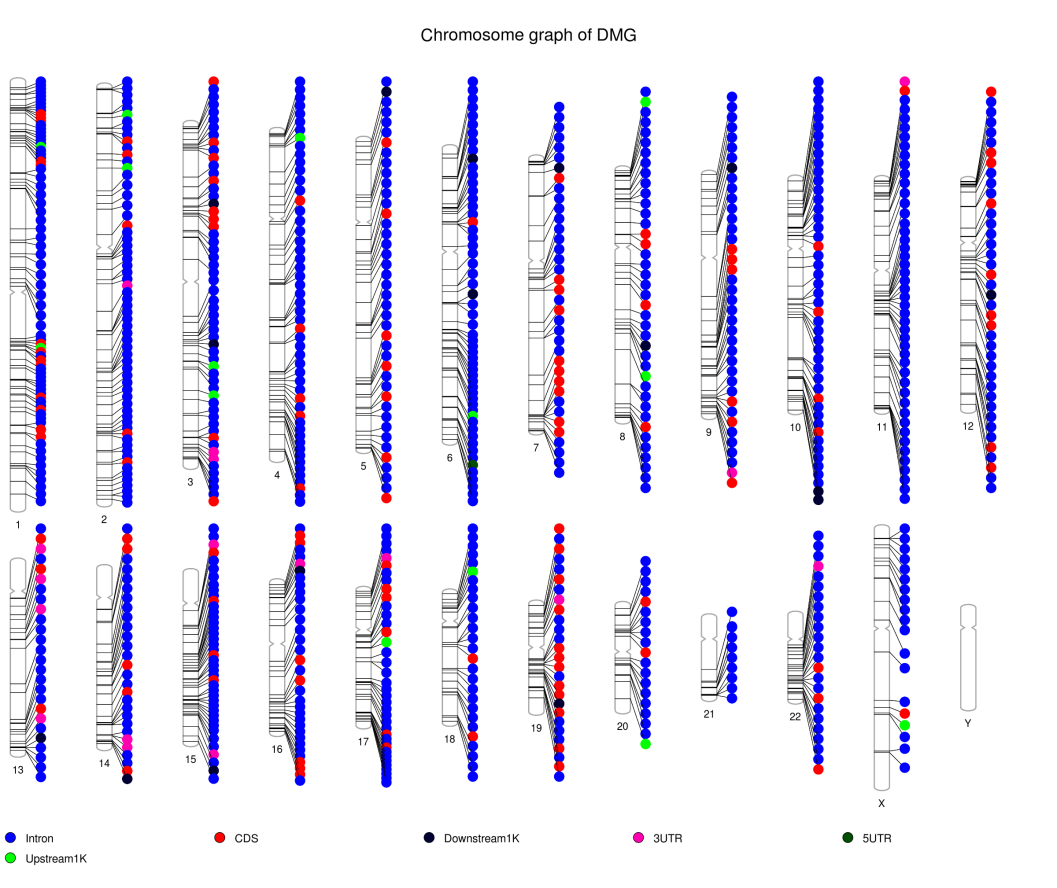


**Supplementary Figure 1.** Chromosome graph of differentially methylated regions beween PCOS and control GCs. Different colors represent different types of DMR locus.


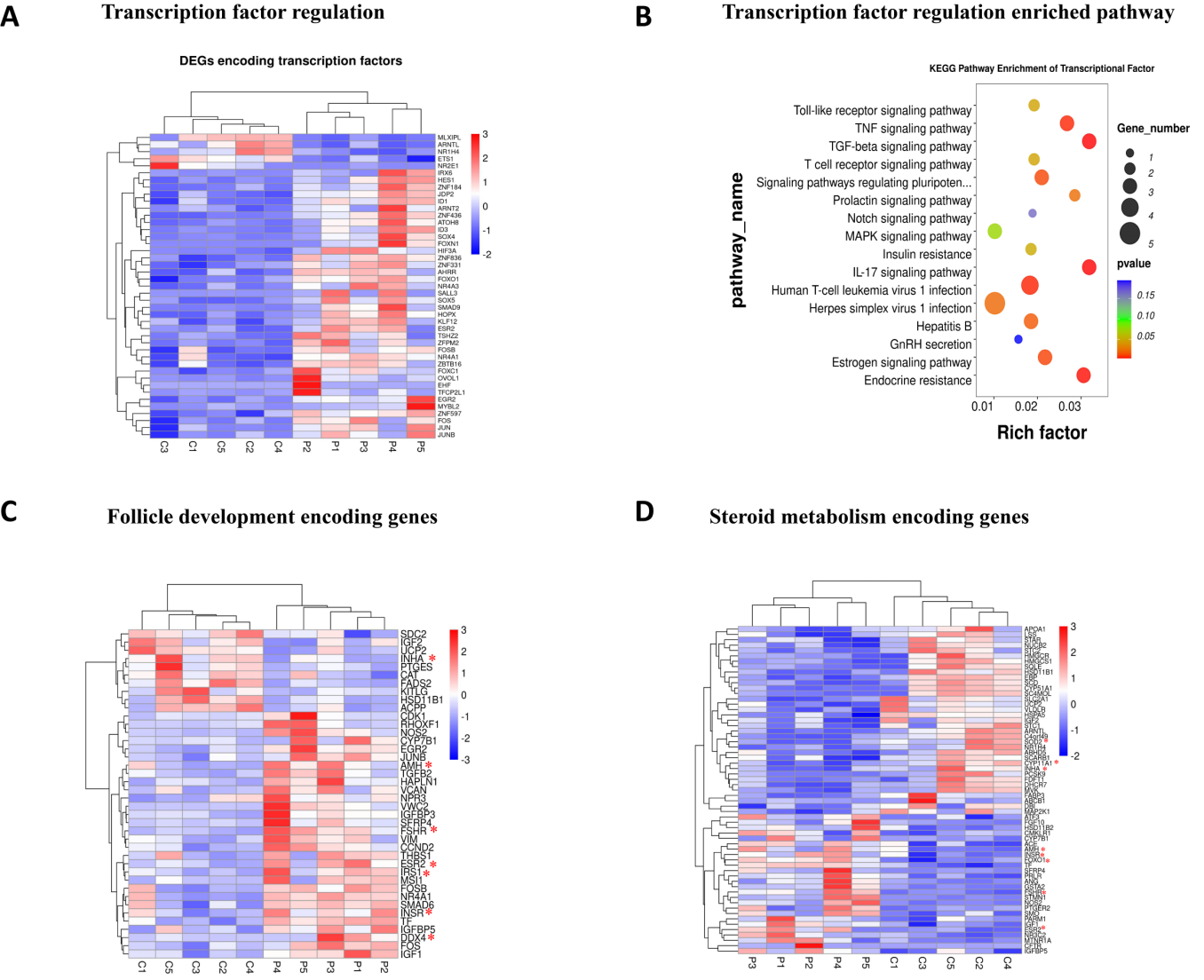


**Supplementary Figure 2.** Figure S2. Specific pathway analysis of differentially expressed genes. (A) Heatmap of DEGs associated with transcription factor regulation. (B) KEGG pathways of DEGs associated with transcription factor regulation. (C) Heatmap of DEGs associated with follicle development. (D) Heatmap of DEGs associated with steroid metabolism.
